# Force Optimization of Elongated Undulating Fin Robot Using Improved PSO-Based CPG

**DOI:** 10.1155/2022/2763865

**Published:** 2022-03-09

**Authors:** Van Dong Nguyen, Quang Duy Tran, Quoc Tuan Vu, Van Tu Duong, Huy Hung Nguyen, Thi Thom Hoang, Tan Tien Nguyen

**Affiliations:** ^1^National Key Laboratory of Digital Control and System Engineering (DCSELab), Ho Chi Minh City University of Technology (HCMUT), 268 Ly Thuong Kiet Street, District 10, Ho Chi Minh City, Vietnam; ^2^Faculty of Electronics and Telecommunication, Saigon University, Ho Chi Minh City, Vietnam; ^3^Department of Electronic & Electrical Engineering, NhaTrang University, Nha Trang, Vietnam

## Abstract

Biorobotic fishes have a huge impact on the development of underwater devices due to both fast swimming speed and great maneuverability. In this paper, an enhanced CPG model is investigated for locomotion control of an elongated undulating fin robot inspired by black knife fish. The proposed CPG network includes sixteen coupled Hopf oscillators for gait generation to mimic fishlike swimming. Furthermore, an enhanced particle swarm optimization (PSO), called differential particle swarm optimization (D-PSO), is introduced to find a set of optimal parameters of the modified CPG network. The proposed D-PSO-based CPG network is not only able to increase the thrust force in order to make the faster swimming speed but also avoid the local maxima for the enhanced propulsive performance of the undulating fin robot. Additionally, a comparison of D-PSO with the traditional PSO and genetic algorithm (GA) has been performed in tuning the parametric values of the CPG model to prove the superiority of the introduced method. The D-PSO-based optimization technique has been tested on the actual undulating fin robot with sixteen fin-rays. The obtained results show that the average propulsive force of the untested material is risen 5.92%, as compared to the straight CPG model.

## 1. Introduction

Robotic technologies are attracting significant attention from researchers, especially in the fields of outer space and ocean exploration that are difficult for humans to access. For example, in the area of ocean science, autonomous underwater vehicle (AUV) has been strongly developed by using different propulsion mechanisms such as jets, axial propellers, and body or fin [1–3]. Because of low noise, high maneuverability, and rapid speed, bionic fish robots with fin propulsion systems have been widely applied for a large number of underwater devices. Recently, many researchers have investigated to improve the motion performance of biorobotic fishes using fin-ray as a propulsion [4, 5], in which dynamic modeling, locomotion control, and optimization are mainly focused.

In motion control, the rhythmic movements of biorobotic fishes are produced by central pattern generator (CPG) networks [6–8]. Biological CPG serves neural networks that can generate patterned neural without any periodical inputs obtained by higher control centers or the feedback signal from sensors [9]. However, using some differential equations with various imprecise parameters, selecting a set of CPG characteristic parameters for improved performance is necessary.

To simulate the CPG behavior, many researchers have established some of the different mathematical models to seek the parametric values of CPG to obtain the desired oscillation profiles. These researchers have attempted to adopt the CPG model to generate the rhythmic movements of a species of elongated undulating fin robot, which comprises multiadjacent fin-rays interconnected to a flexible membrane. The authors in reference [10] proposed a CPG integrated with PID to establish a motion controller for a prototype of the fish robot. In paper [11], a CPG network comprising ten Matsuoka oscillators is offered to generate rhythmic signals. In 2012, Zhou et al. introduced a CPG model using two motors to drive two pectoral fins for a manta ray robot [12]. Due to the slow response time, an improved CPG using a genetic algorithm is performed for the thrust generation of the fish robot [13]. In paper [14], a CPG model is adopted to achieve the undulating motion pattern for finding the critical factor, which affects the propulsion. Although the above mathematical models have been successfully applied for establishing a CPG-based motion controller, enhancing the propulsive force of the robotic fish using a CPG network is still a significant challenge. To overcome this problem, optimization algorithms have been performed for parameter selection by some researchers. A Hopf oscillator-based CPG network performed the parameter synthesis subjecting to some learning rules to obtain the desired swimming pattern [15]. Meanwhile, the modified CPG in paper [16] can produce different locomotion patterns of an actual fish by combining Andronov–Hopf oscillators and an artificial neural network (ANN). Recently, heuristic search has been widely applied for tuning the parameters of the CPG network. In paper [17], the genetic algorithm (GA) is used for the rhythmic generation based on CPG models by establishing the weight values of the coordination between oscillators. The authors in references [18, 19] both use particle swarm optimization (PSO) to find the optimal characteristic parameters of the Hopf oscillator-based CPG for improved propulsive performance. Although these metaheuristic algorithms are well resulted in seeking the CPG parameters, they are often trapped in local optima. In this brief, a new differential particle swarm optimization (D-PSO) is investigated to improve optimization problems.

A brief of our main contributions can be expressed as follows: At first, a CPG using the chain topology of sixteen coupled Hopf oscillators is offered to generate fishlike rhythmic movements. Following this, the improved D-PSO is exploited to optimize the amplitude values of the CPG network to increase the average propulsive force of the undulating fin robot to make a faster movement. Finally, the obtained result of CPG parameter synthesis using the different optimization methods, including D-PSO, PSO, and GA, is implemented to prove the superiority of the proposed D-PSO algorithm.

The rest of this study is categorized into four parts. In Section 2, material and research methods, including dynamic modeling and the modified CPG-based locomotion control, are presented.  Section 3 presents the optimization method of the CPG model using the variant of PSO. Simulation results and discussions are illustrated in Section 4. Finally, Section 5 gives a conclusion.

## 2. Materials and Methods

### 2.1. Dynamic Modeling of Elongated Undulating Fin Robot

**Figure 1:**
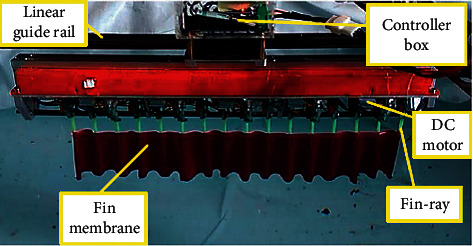
Actual experiment model.

The elongated undulating fin robot is composed of sixteen links and a flexible membrane mounted on the straight axis base of underwater vehicles. Each link includes an RC servo motor and an oblique fin ray that can rotate around a joint, as illustrated in Figure 1. The distance between the adjacent link is 32 mm, and the length of the fin-ray is 150 mm. The elongated undulating fin generates a sinusoidal oscillatory whose amplitude envelope is defined by sine wave trajectory to perform forward moving. The sinusoidal oscillatory is propagated along to the fin from the anterior to the posterior and is determined by the following equation:

(1) $$\begin{array}{c} \theta^{i}\left(t\right)=\theta_{m\max}^{i}sin\left(2\pi ft+\phi_{i}\right), \end{array}$$

where *θ*^*i*^ is the sway angle of the *i*^th^ fin-ray at the time *t*; *θ*^*i*^_max_ is the maximum sway angle for each fin-ray; *f* is the oscillatory frequency; ϕ_*i*_ is the phase difference angle between two adjacent fin-rays called the phase lag angle.

**Figure 2:**
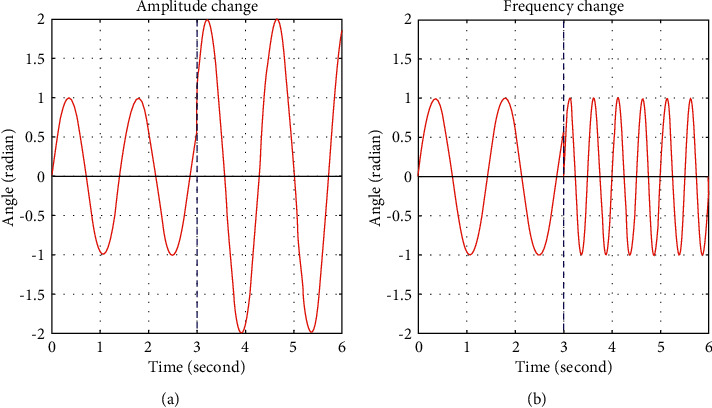
Output of sinusoidal equation under the change of amplitude and frequency.

It can be observed from Figure 2 that there is a discontinuity in the sinusoidal oscillatory trajectory at the arbitrary time *t*^*∗*^ corresponding to the change of the amplitude envelope (Figure 2(a)) and the oscillatory frequency (Figure 2(b)). In this paper, a modified CPG is introduced to obtain flexible fishlike locomotion in the following section.

### 2.2. Hopf Oscillator-Based CPG Model

**Figure 3:**
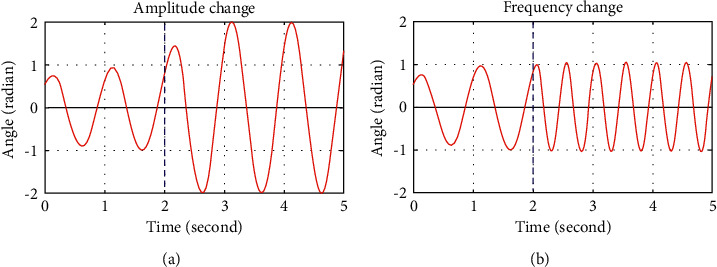
Output of Hopf oscillator under the change of amplitude and frequency.

CPGs are known as neural neuron whose oscillator is spiked after each cycle, resulting from mutant inhibition of neurons. To construct the CPG model, various research studies have been employed, including Wilson–Cowan, Kuramoto, Matsuoka, amplitude-controlled phase, Rowat–Selverston, and Hopf. The Hopf oscillator is known as the most promising model for simulating the CPG behavior. The Hopf oscillator can generate the continuous sinusoidal oscillation even when there is a sudden change of both amplitude envelope and oscillatory frequency at the arbitrary time *t*^*∗*^, as shown in Figure 3, demonstrating its superiority as compared to the traditional sinusoidal generator. Furthermore, the Hopf oscillator has a direct relationship between model parameters and output variables in nonlinear oscillators. Therefore, this paper chooses the Hopf oscillator as the best tool for building the CPG network.

The dynamic of the Hopf oscillator is defined as follows:

(2) $$\begin{array}{c} \left\{\begin{array}{c} \overset{\cdot }{p_{i}\left(t\right)}=k\left(A_{i}^{2}-p_{i}^{2}\left(t\right)-q_{i}^{2}\left(t\right)\right)p_{i}\left(t\right)-2\pi fq_{i}\left(t\right),\\ \overset{\cdot }{q_{i}\left(t\right)}=k\left(A_{i}^{2}-p_{i}^{2}\left(t\right)-q_{i}^{2}\left(t\right)\right)q_{i}\left(t\right)-2\pi fp_{i}\left(t\right), \end{array}\right. \end{array}$$

where *p* and *q* present the state variables of the nonlinear oscillator, A and *f* are known as the amplitude and the intrinsic frequency of the oscillator, and *k* is the convergence speed. Due to using a harmonic limit cycle, in the steady-state condition, the Hopf oscillator is expressed as follows:

(3) $$\begin{array}{c} \left\{\begin{array}{c} \overset{\cdot }{p_{\infty }\left(t\right)}=kA\left(c\text{os}2\pi \text{t}+\varphi_{0}\right),\\ \overset{\cdot }{q_{\infty }\left(t\right)}=kA\left(\sin\;2\pi \text{t}+\varphi_{0}\right), \end{array}\right. \end{array}$$

where *φ*_0_ denotes the initial phase of the oscillation. From (3), we can clearly see that it is easy to adjust the convergence speed of the Hopf oscillator according to the change of the parameter *k*. In other words, the Hopf oscillator gives a faster convergence speed to the limit cycle corresponding to an increase of *k*. Furthermore, due to the limit cycle being structurally stable, the general behavior of the system remains constant with a small perturbation on the oscillator or return to the original orbit in case of larger perturbation.

### 2.3. A Modified CPG

As mentioned above, the structure of the elongated undulating fin robot includes a set of joints in which a CPG unit regulates each joint. In other words, each CPG output corresponding to a state variable *p* of the Hopf oscillator will define the sway angle around a joint. To generate the rhythmic oscillatory for swimming pattern, it is necessary to link these oscillators together through a proper coupling configuration known as an additive perturbation on the nonlinear oscillatory generator. The perturbed oscillator can be presented as follows:

(4) $$\begin{array}{c} {\dot{U}}_{i}=F\left(U_{i}\right)+Y_{i}=\left[\begin{array}{c} k\left(A_{i}-p_{i}^{2}-q_{i}^{2}\right)p_{i}-2\pi fq_{i}\\ k\left(A_{i}-p_{i}^{2}-q_{i}^{2}\right)q_{i}+2\pi fp_{i} \end{array}\right]+\left[\begin{array}{c} y_{\left(p,i\right)}\\ y_{\left(q,i\right)} \end{array}\right], \end{array}$$

where $\dot{U_{i}}$ is the state vector of the *i*^th^ oscillatory generator; *F*(*U*_*i*_) is a nonlinear function; *Y*_*i*_ is a perturbation vector.

**Figure 4:**
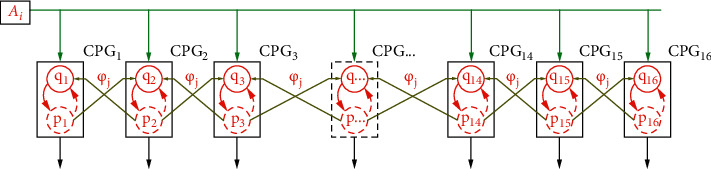
Structure of the CPG network with bidirectional couplings.

It is noted that the amplitude values of an oscillator are affected by the perturbation signal, and hence each oscillator can be coordinated with another through a perturbation to recognize the change of amplitude and maintain the stable frequency and phase. There are various proposed coupling structures, including ring, chain, star, and full topologies. In the paper, however, the chain structure with adjacent neighbor connections is chosen to obtain the oscillatory coordination of fin-rays. It means a chain of CPGs will use serially connected joints for the gait generation of the elongated undulating robot. Furthermore, the proposed CPG structure uses bidirectional couplings that can be influenced by both anterior and posterior adjacent fin-rays, as shown in Figure 4.

It is clear from Figure 4 that one oscillator is only affected from the nearest neighbor oscillator though the perturbation, so the perturbed oscillator equation can be rewritten as follows:

(5) $$\begin{array}{c} {\dot{U}}_{i}=\left[\begin{array}{c} k\left(A_{i}-p_{i}^{2}-q_{i}^{2}\right)p_{i}-2\pi fq_{i}\\ k\left(A_{i}-p_{i}^{2}-q_{i}^{2}\right)q_{i}+2\pi fp_{i} \end{array}\right]+\left[\begin{array}{c} 0\\ \beta \left(p_{i-1}\sin\;\varphi_{j}+q_{i-1}\text{cos }\varphi_{j}-p_{i+1}\sin\;\varphi_{j}+q_{i+1}\text{cos }\varphi_{j}\right) \end{array}\right]. \end{array}$$

From the above equation, we can see that the modified CPG network can generate the rhythmic movement of the undulating fin robot by adjusting the amplitude values *A*_*i*_ to obtain the different propulsive forces. In this research, a novel variant of PSO is introduced to optimize the oscillatory amplitudes of the modified CPG model for the purpose of improving the swimming ability of the undulating fin robot.

## 3. Developed PSO-Based CPG Optimization

### 3.1. D-PSO

PSO simulates the social behavior of some species such as flocks of birds and schools of fish to exploit the best solution in a nonlinear search space. Each particle updates its next positions by three following values, including the current velocity of that particle, its previous best position (*P*_best_), and the best fitness value (*G*_best_). The theory of PSO can be consulted in detail in references [20–22]. However, the main disadvantage of traditional PSO is easy to be fallen into the local extreme domain. To overcome this problem, a new version of PSO, namely D-PSO, is developed in this study to enhance the optimization performance.

D-PSO is performed by adding a feature into the velocity equation of PSO. The additive feature is known as the best position of an individual that is randomly taken in the population. The velocity equation of particle after adding one more term can be written as follows:

(6) $$\begin{array}{c} V_{i,j}^{ite+1}=w\times V_{i,j}^{ite}+c_{1}r_{1}\left(P_{\text{bes}ti,j}^{ite}-X_{i,j}^{ite}\right)+c_{2}r_{2}\left(G_{bestj}^{ite}-X_{i,j}^{ite}\right)+c_{3}r_{3}\left(X_{h,j}^{ite}-X_{i,j}^{ite}\right), \end{array}$$

(7) $$\begin{array}{c} X_{i,j}^{ite+1}=X_{i,j}^{ite}+V_{i,j}^{ite+1}. \end{array}$$

**Figure 5:**
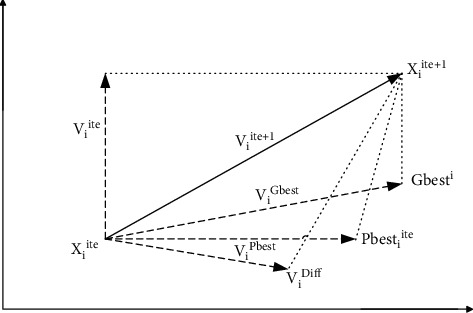
Diagram of the updating process of the particle *i* at the iteration *ite*.

In the above equation, *c*_3_ denotes the scaling factor and *r*_3_ is selected randomly in the range of [0, 1], whereas *h* is a varying parameter (from 1 to *N*) that denotes the expert particle corresponding to the target particle *p*. The new position updating process of D-PSO is shown in Figure 5.

It is clear from Figure 5 that *P*_*besti*,*j*_^*ite*^ is the *j*^th^ best component of the *i*^th^ particle and *G*_*j*_^*ite*^ is the *j*^th^ component of the best particle in the swarm at the iteration *ite*. It is noted that *V*_*i*_^*Diff*^ is the additive feature in the velocity equation whose randomly scaled difference and another particle make them avoid the local optima points.

### 3.2. Application of D-PSO to CPG Model

The performance of the CPG model is susceptible to the value of amplitude, and hence it is necessary to seek the best parametric values for the purpose of improving the propulsive performance. In this section, a novel D-PSO is applied to obtain a set of optimum amplitudes by maximizing the average propulsive force called the objective function. The optimization problem of the CPG model can be posed in the following way:

(8) $$\begin{array}{c} \text{Maximizing}:F=\frac{1}{\Delta t}\int_{0}^{\Delta t}F\left(t\right)dt,\\ \text{Subject to}:\left\{\begin{array}{c} A_{\min}\leq A\leq A_{\max},\\ A_{i}<A_{i+1},i=1÷16. \end{array}\right. \end{array}$$

The developed D-PSO-based CPG optimization problem is performed as follows:

(1) Select some parameters of D-PSO, including *w, c*_1_*, c*_2_, and *c*_3_
(2) The initial positions and velocities of each individual in the swarm are selected by random values
(3) Initialize oscillation amplitudes of the CPG model within their ranges
(4) Call the Hopf oscillator-based CPG model
(5) The fitness function of each individual is evaluated by
  (9) $$\begin{array}{c} FN_{i}^{ite}=\;f\left(X_{i}^{ite}\right),\forall i. \end{array}$$
  The particle having the best position is indexed as *p*, and hence the personal experience and the overall experience are selected as follows:
  (10) $$\begin{array}{c} P_{best}^{ite}=\;X_{i}^{ite},\;and\;G_{best}^{ite}=\;X_{p}^{ite}. \end{array}$$
(6) Initialize the iteration at *ite* *=* 1
(7) The velocity and position of each individual are updated by (6) and (7)
(8) The updated fitness function of each particle is re-evaluated:
*FN*_*i*_^*ite*+1^= *f*(*X*_*i*_^*ite*+1^), *∀i* and indexing for the particle with the best position as *q*
The personal experience and the overall experience of the swarm are updated as follows:
  (11) $$\begin{array}{c} \text{If }FN_{i}^{ite+1}<\;FN_{i}^{ite}\;then\;P_{besti}^{ite+1}=X_{i}^{ite+1}\;else\;P_{besti}^{ite+1}=\;P_{besti}^{ite},\\ \text{If }FN_{q}^{ite+1}<F_{b}^{k}\;then\;G_{best}^{ite+1}<P_{besti}^{ite+1}\;and\;select\text{ p}=\text{q }else\;G_{best}^{ite+1}<G_{best}^{ite}, \end{array}$$
(9) If *ite* *<* *Maxite*, then ite = ite + 1 and goto the step 7 else goto the step 10
(10) The optimal parameters of CPG are obtained as *G*_*best*_^*ite*^, and hence the maximum thrust force is defined.

**Figure 6:**
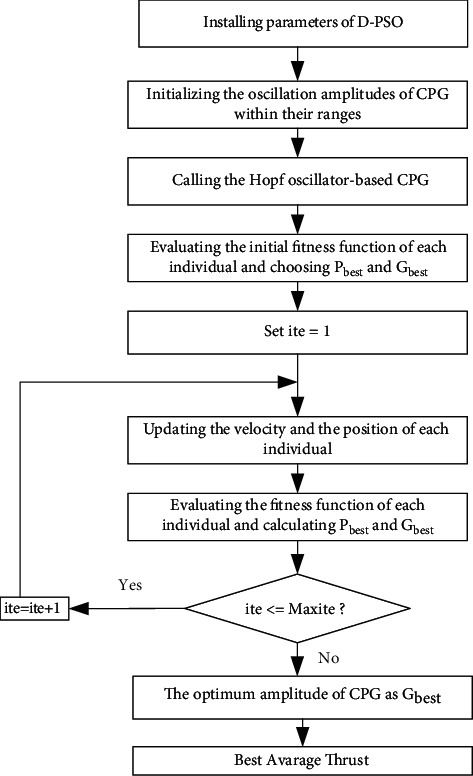
Flowchart of the proposed approach.

A detailed flowchart of the proposed D-PSO-based CPG model considering the above steps is shown in Figure 6.

## 4. Test Results and Discussion

**Figure 7:**
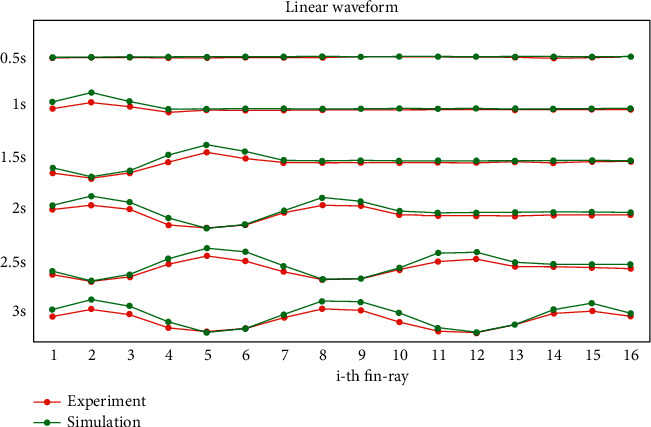
The output of the real CPG model.

The proposed D-PSO-based CPG optimization method is performed both on the simulation model in MATLAB and during an experiment with the real elongated undulating fin. For the simulation test, a dynamic analysis of the elongated undulating fin robot that was presented in our previous works [23, 24] is adopted to evaluate the thrust force produced by the undulating fin-ray. An actual sixteen fin-ray robot with a size of 775 mm long, 90 m wide, and 290 m deep was designed for the experimental purpose. The oscillatory frequency is chosen at 1 Hz, and the coupling strength is 0.8. The actual testing result of the output signals of CPG is illustrated in Figure 7.

**Figure 8:**
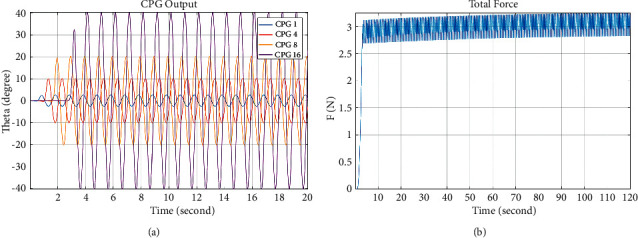
Simulation results with the random values of amplitude. (a). The CPG outputs. (b) The characteristic curve of average thrust.

In the term of simulation, each joint of the undulating fin is driven by the CPG unit, whose amplitude values are chosen in the range of [0, 40] degree based on the actual mechanical structure. According to a set of parameters, as given in Table 1, the output of CPG model is presented in Figure 8(a) and the thrust curve obtained by the Hopf-based CPG outputs is illustrated in Figure 8(b). In the term of simulation, each joint of the undulating fin is driven by the CPG unit, whose amplitude values are chosen in the range of [2.5, 40] degree based on the actual mechanical structure. According to a set of parameters, as given in Table 1, the output of CPG model is presented in Figure 8(a) and the thrust curve obtained by the Hopf-based CPG outputs is illustrated in Figure 8(b).

It can be seen from Figure 8(b) that the average push force of sixteen fin-ray system can reach 2.92 N; however, there is an oscillation around a steady state.

To reach the best performance of CPG units, a new D-PSO algorithm is governed for the first time to obtain the maximum average propulsive force. However, the D-PSO only achieves the best optimum results once the appropriate parameters are selected in order to increase the converge speed as well as avoid trapping the local optimal point. After ten times repeated running, we obtained the parameters of D-PSO corresponding to the population size of ten particles as follows:

1. The weight coefficient (*w*) is in the range of [0.1, 0.9]
2. The constant factors (*c*_1_ and *c*_2_) are set as 2
3. The scaling factor (*c*_3_) is selected as 0.04
4. The updating procedure is employed in 100 iterations

### 4.1. Testing the D-PSO Algorithm on the Basic Math Function

To prove the feasibility of the developed D-PSO technique in finding the extremum of functions, some of basic math functions given have been initially tested, as shown in Table 2. It is noted that D-PSO is performed using the above selected parameters corresponding the population size of 10 particles.

It is clear from Table 2 that the proposed D-PSO technique is the ability to optimize the basic five math functions successfully. For the population size of ten particles, the D-PSO achieves the least value of mean square error (MSE) of five math functions as 8.11E − 05, 0.000928, 0.000389, 3.37E − 15, and 2.80E − 12, respectively.

### 4.2. Testing the D-PSO Algorithm on the Modified CPG Network

Table 3 gives the average propulsive force of the undulating fin with the dynamic model driven by Hopf oscillator-based CPG unit using both with and without D-PSO optimization.

**Figure 9:**
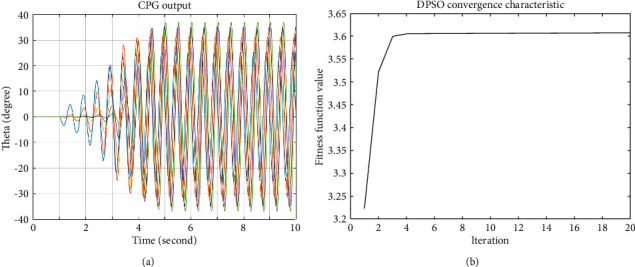
Simulation results with D-PSO-based CPG. (a). The outputs of D-PSO-based CPG. (b) The average thrust force.

It can be observed from Table 3 that the average push force before optimization with the random chosen parameters is 0.52 N, whereas this value is increased to 3.6 N after using the D-PSO-based CPG. According to the constant values of the intrinsic frequency of 1 Hz, the best amplitude parameters of A_1_–A_16_ is given in Table 3. Furthermore, the D-PSO-based CPG output and the corresponding average thrust force are shown in Figures 9(a) and 9(b), respectively.

Table 4 shows a comparison of D-PSO with the traditional PSO and GA to prove the superiority of the developed method in this study. The parameter selection of PSO and GA is also performed through running repeatedly many times corresponding to the different population sizes. As a result, the best acceleration coefficients are both selected as 2.0 for PSO, while GA uses the best crossover probability as 0.75 and the best mutation fraction as 0.015.

**Figure 10:**
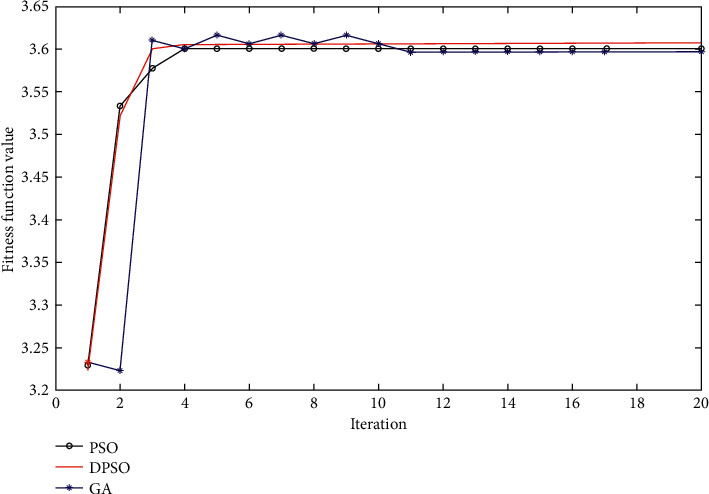
The convergence characteristic of some CPG optimization techniques.

From Table 4, we can see that the optimum results obtained by the D-PSO are better than those obtained by any other CPG parameter optimizers. The average thrust is 3.60 N in the case of using the proposed D-PSO, while the values of force only reach 3.58 N and 3.57 N by PSO and GA, respectively. Moreover, Figure 10 illustrates the convergence characteristic of average thrust obtained using three different optimization techniques, including D-PSO, PSO, and GA. We can see clearly from Figure 10 that the convergence time of the proposed D-PSO is faster than that of PSO and GA. The D-PSO only takes 2.3 iterations to converge; meanwhile, these values are 3.9 iterations for PSO and 10.5 iterations for GA, respectively. It can be further seen from Figure 10 that there are two steps in the convergence characteristic of PSO and GA, so it is easy for these two optimization methods to get trapping the local maxima. This problem has been solved by using the proposed D-PSO, whose optimization result only shows one step. It means the D-PSO is the ability to achieve the global best position better than the others.

## 5. Conclusions

In this paper, the locomotion control and optimization task have been successfully performed on the elongated undulating fin robot inspired by the black knife fish. A bidirectionally coupled sixteen Hopf oscillator-based CPG network is investigated to create rhythmic movement as a perfect paradigm for robotic locomotion. In addition, a novel variant of PSO, namely D-PSO, has been developed to obtain a set of optimum amplitudes of the modified CPG network for the purpose of maximizing the average propulsive force. In brief, the new contributions in our study can be drawn as follows:

1. In the term of motion control scheme, the CPG based on coupled Hopf oscillators not only generates the desired rhythm for the undulating fin robot but also removes the effect of radial interference on the oscillatory amplitude.
2. The proposed D-PSO algorithm is the capacity to successfully solving the basic math functions and effectively improving the average thrust of the untested undulating fin fish obtained from the output signals of modified CPG.
3. The novel D-PSO algorithm gives the fastest propulsive force as compared to PSO and GA. Moreover, the superiority of the proposed method is presented in the ability to avoid the local maxima in order to increase the accuracy of the optimization process.

## Figures and Tables

**Table 1:** Parameters of CPG network.

*f* (Hz)	*φ* (°)	*A*_1_	*A*_2_	*A*_3_	*A*_4_	*A*_5_	*A*_6_	*A*_7_	*A*_8_	*A*_9_	*A*_10_	*A*_11_	*A*_12_	*A*_13_	*A*_14_	*A*_15_	*A*_16_
**1**	−60°	2.5	5.0	7.5	10.0	12.5	15.0	17.5	20.0	22.5	25.0	27.5	30.0	32.5	35.0	37.5	40.0

**Table 2:** The tested five math functions.

Function name	Equation	Variable range	Extreme value	MSE
Beale	$\begin{array}{c} F_{1}={\left(1.5-u_{1}-u_{1}u_{2}\right)}^{2}+{\left(2.25-u_{1}-u_{1}u_{2}^{2}\right)}^{2}+{\left(2.625-u_{1}-u_{1}u_{2}^{3}\right)}^{2} \end{array}$	[−10, 10]	*F*(3,0.5)=0	8.11E − 05
Levi	*F*_2_= sin^2^(3*πu*_1_)+(*u*_1_^2^ − 1)^2^(1+ sin^2^(3*πu*_2_))+(*u*_2_^2^ − 1)^2^(1+ sin^2^(3*πu*_2_))	[−10, 10]	*F*(1,1)=0	0.000928
Booth	*F*_3_=(*u*_1_+2*u*_2_ − 7)^2^+(2*u*_1_+*u*_2_ − 5)^2^	[−10, 10]	*F*(3,1)=0	0.000389
Sphere	$F_{4}=\sum_{i=1}^{n}u_{i}^{2}$	[−20, 20]	*F*(0,0, ..., 0)=0	3.37E − 15
Ackley	$F_{5}=20+e-20\;\exp\left(-0.2\sqrt{\frac{1}{n}\sum_{i=1}^{n}u_{i}^{2}}\right)-\exp\left(\frac{1}{n}\sum_{i=1}^{n}\cos\left(2\pi u_{i}\right)\right)$	[−20, 20]	*F*(0,0, ...0)=0	2.80E − 12

**Table 3:** Optimization results of CPG model with/without D-PSO algorithm.

Model	*A*_1_	*A*_2_	*A*_3_	*A*_4_	*A*_5_	*A*_6_	*A*_7_	*A*_8_	*A*_9_	*A*_10_	*A*_11_	*A*_12_	*A*_13_	*A*_14_	*A*_15_	*A*_16_	F(N)
Straight CPG	2.5	5.0	7.5	10.0	12.5	15.0	17.5	20.0	22.5	25.0	27.5	30.0	32.5	35.0	37.5	40.0	2.92
D-PSO CPG	2.324	5.278	12.698	16.508	19.002	20.221	21	22	24	25.249	28	30.205	32.113	36	38	40	3.60

*Note*. *v* denotes the average propulsive speed, and *t* is the convergence time.

**Table 4:** Optimization results of CPG model using different metaheuristic algorithms.

Model	*A*_1_	*A*_2_	*A*_3_	*A*_4_	*A*_5_	*A*_6_	*A*_7_	*A*_8_	*A*_9_	*A*_10_	*A*_11_	*A*_12_	*A*_13_	*A*_14_	*A*_15_	*A*_16_	F(N)
GA-CPG	1.362	5.048	8.911	11.754	12.495	17.484	22.489	23.108	23.617	24.625	26.500	33.765	32.438	35.679	38.473	39.049	3.57
PSO CPG	1	5.818	7.125	11	13.988	16.747	23.977	24.217	25.234	26	27	35.325	36.763	37.358	38.946	39.403	3.58
D-PSO CPG	2.324	5.278	12.698	16.508	19.002	20.221	21	22	24	25.249	28	30.205	32.113	36	38	40	3.60

## Data Availability

No data were used to support this study.
